# Effect of Normal and Rubberized Concrete Properties on the Behavior of RC Columns Strengthened with EB CFRP Laminates and Welded Wire Mesh under Static Axial Loading

**DOI:** 10.3390/polym14245351

**Published:** 2022-12-07

**Authors:** Ibrahim A. Sharaky, Ahmed S. Elamary, Yasir M. Alharthi, Ayman Abdo

**Affiliations:** 1 Civil Engineering Department, College of Engineering, Taif University, P.O. Box 11099, Taif 21944, Saudi Arabia; 2 Structural Engineering Department, Faculty of Engineering, Zagazig University, Zagazig P.O. Box 44519, Egypt

**Keywords:** crumb rubber, fiber reinforced polymer, externally bonded, CFRP laminates, welded wire mesh, recycled steel fiber

## Abstract

The huge amounts of old and damaged tires spread worldwide has caused many complex environmental risks. The old tires have been converted to crumb rubber (CR) and tire recycled steel fiber (RSF) to facilitate their use. This study used CR to partially replace natural sand in reinforced (RC) columns. Externally bonded (EB) carbon-fiber-reinforced polymer (CFRP) laminates, welded wire mesh (WWM), and RSF were used to enhance the axial behavior of the tested columns to overcome the concrete deficiencies resulting from the inclusion of the CR instead of natural sand. Eighteen columns were prepared and tested to discuss the effects of strengthening type, CR content, RSF, and strengthening area on the axial behavior of the RC columns. Certain columns were internally reinforced with WWM, while others were externally strengthened with EB CFRP laminates. Partially or fully EB CFRP laminates were used to strengthen the columns. Moreover, one column was cast with NC and 0.2% RSF to investigate the role of RSF in confining the column. The results demonstrated a concrete strength reduction for the rubberized concrete (CRC) as the CR content increased. Conversely, the strengthened columns experienced higher load capacities than the corresponding un-strengthened ones cast with the same concrete mix. Moreover, adding 2% RSF to the NC mix could enhance the column capacity, although it decreased the concrete strength. Furthermore, using two CFRP layers increased the load capacity and ductility of the strengthened columns. The strengthened column cast with 50% CR showed the highest load efficiency (334.3% compared to the un-strengthened one).

## 1. Introduction

The annual production of car tires creates huge amounts of old and damaged tires, which cause many complex environmental risks. Researchers have been preoccupied with the possibility of using old tire components such as crumb rubber (CR) in various fields to diminish their environmental impact (EI) [1]. Moreover, the increasing demand for concrete consumes the natural aggregate and threatens natural resources [2]. In the engineering and construction fields, using CR to substitute the natural concrete component to produce rubberized concrete (CRC) reduces the amount of disposed tires and accordingly preserve natural resources [3,4,5,6,7]. The CRC could improve the earthquake resistance by dissipating energy, but it reduces the concrete compressive strength [8]. The negative impact of using CR on the concrete’s mechanical properties was additionally stated in [9,10,11,12]. The main reasons for this reduction are the low density of CR compared to the natural aggregate (NA), and the weak bond between the CR and the cement paste. Moreover, the inability of CR to absorb water increases air spaces, causing a fragile matrix and increasing the stress concentration [13,14,15]. Also, the decline in the CRC properties was attributed to crack formation in the interfacial transition region [16,17]. In addition, incorporating excessive CR amounts into concrete (more than 25% of the natural aggregate by volume) adversely affected the environment due to the great energy utilization in CR manufacturing [18].

The fresh and hardened strength factors and the SEM/XRD analysis of high-strength concrete (HSC) incorporating up to 30% CR as a sand replacement were discussed in [19]. When the CR content was 20%, a loss of workability, density, compressive and tensile strengths, and elasticity modulus was observed, but there was a gain in ductility. In [20], CRC was produced by replacing the fine NA with four CR percentages (0%, 5%, 15%, and 25%). The tests showed that using CR worsened all the measured properties of CRC. Several researchers have developed curing methods for concrete containing CR to enhance brick and concrete mechanical properties [3,21,22,23,24,25,26,27,28]. Waste quarry dust (WQD) has been used as a pretreatment for CR [29]. Four replacement ratios of sand (5%, 10%, 15%, and 20%) were used with untreated CR and WQD-treated CR. At 20% CR content, the treated CR enhanced the CRC properties in contrast to the untreated CR. Moreover, in [27] silica fume self-consolidating CRC (SLFSCRC) mixes were adjusted to increase the CR content and reduce CRC strength. The silica fume (SF) could enhance the CRC strength even after using 25% CR. Pre-coating the CR surface with resin and micro-silica improved CRC properties [25]. Double pre-coated rubber techniques modified the CRC compressive and flexural strengths and the young’s modulus by 60%, 30%, and 28%, respectively. The influence of pretreatment CR on concrete properties was also examined in [21,22,23,24]. The CRC could be applied to earthquake-prone structures as it had a high energy dissipation capacity to reduce the vibration amplitude [5,30,31,32].

Because CR diminishes the compressive strength of concrete, confinement to the outer surface of the concrete, especially in the columns, was proposed. CRC confinement could recover its strength loss. Negative confinement varies with concrete deformation where the FRP wrap is exclusively homogeneous on circular shafts under a concentric weight [28,33,34,35,36,37]. Actively trapped columns should also be studied to examine the behavior of a steel-entrapped column under continuous lateral stress after the lateral reinforcement yields. In [38], CRC short columns under varied exposure circumstances were examined. Three concrete mixes with CR content (0, 10, and 20%) to replace fine NA were performed. As the CR content increased, the mechanical characteristics and durability of the concrete and the column load capacities were reduced. In [39], CFRP wrapping and a steel jacket were used to reinforce the columns. Compared to the standard columns, 90% to 128% load capacity improvement was achieved for columns strengthened with CFRP wrapping and a steel jacket. Furthermore, the ductility was enhanced by strengthening procedures from 3% to 24.3%. Columns with lower concrete compressive strengths were more noticeably impacted by strengthening. Conversely, the axial behavior of square and circular concrete columns confined with CFRP sheets and WWM steel confinement at extreme temperatures was examined [40]. All columns lost their axial capacity and initial stiffness when heated. The WWM confinement around elevated-temperature circular and square columns increased strength and stiffness more than the CFRP strips. Additional data about the effect of CRC on columns’ behavior and capacity under several loading conditions were reported in [41,42].

The combination of CFRP confinemen” and’ferrocement was experimentally investigated in strengthening RC columns with different column cross-sectional shapes and confinement amounts [43]. The results show that ferrocement is highly effective if combined with CFRP and it lessens stress concentration, which is generally the reason for the failure of CFRP-reinforced columns. L. Van et al. [44] developed a methodology to predict the failure of carbon-confined FRP (CFRP) columns with different section heights. The effectiveness of the confinement area ratio decreased by increasing structural size. The axial compressive behavior of damaged GFRP-reinforced columns confined with CFRP sheet was studied [45]. It was found that the damaged columns’ ultimate axial strength and displacement capacities increased due to the confinement.

From this review, it is clear that the use of RSF, carbon fiber reinforced polymer (CFRP) as externally bonded (EB) strengthening, and WWM as internal reinforcement to confine the columns cast with CRC, is still limited. Therefore, the behavior of columns cast integrating CR content varying from 0 to 50% replacement of sand was tested. The columns were externally strengthened by EB CFRP laminates or internally reinforced with WWM and RSF. One or two layers of CFRP laminates and WWM were used to strengthen the columns. The CFRP laminates were used as partially or fully EB strengthening. The columns were exposed to axial compression loads until failure. The beam load capacity, contraction, and failure modes were recorded, observed, and compared.

## 2. Experimental Work

### 2.1. Materials

**Figure 1:**
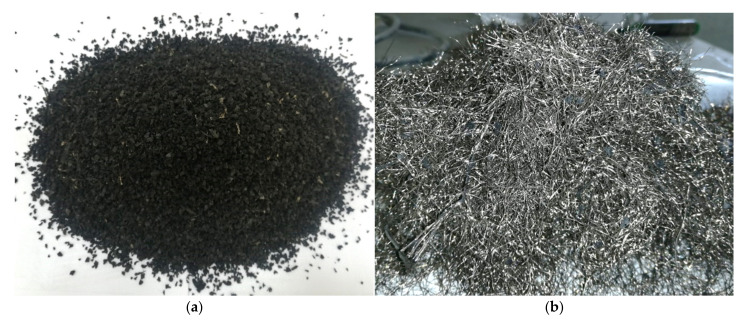
The recycled materials obtained from waste tires. (**a**) Crumb rubber, (**b**) recycled steel bead wires.

**Figure 2:**
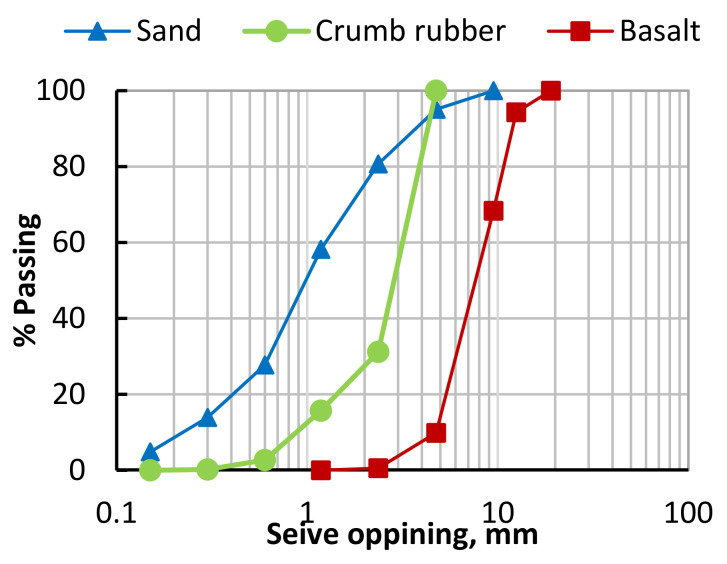
Sieve analysis for the natural aggregates and crumb rubber.

The crumb rubber (CR) powder (Figure 1a) and tire RSF (Figure 1b) were obtained from a local company in Saudi Arabia. The CR had a maximum size of 4.75 mm. The CR’s calculated specific gravity and water absorption were 1.26 and 1.03, respectively, while the fineness modulus was 4.5. The recycled steel bead wires (RSBW) were used as recycled steel fibers (RSF). The RSBW consisted of straight fibers 30 mm to 60 mm in length and 0.2–0.3 mm in diameter. The specific gravity of RSBW, 2.08, was experimentally obtained. The RSBW strength is 2140 Mpa, according to supplier data. Crushed basalt with a maximum nominal size (MNS) of 12.5 mm and natural sand fineness modulus (FM = 3.0) were used as coarse and fine aggregate, respectively. The basalt, CR, and sand grading curves were obtained (Figure 2) according to ASTM C33 Standard [46], while their physical properties are reported in Table 1. All prepared concrete mixes were cast using OPC (initial settling of more than 45 min). The columns’ reinforcement were steel bars with 10 mm diameter, 460 Mpa yield strength, 610 Mpa ultimate strength, and 200 Gpa young’s modulus (from experimental tests).

**Figure 3:**
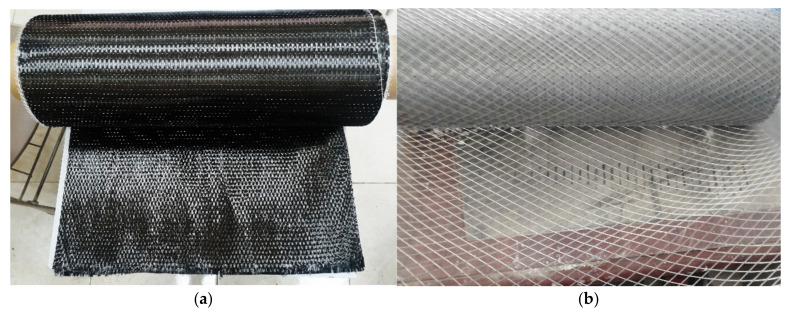
The shape of strengthening materials. (**a**) CFRP laminates, (**b**) welded wire mesh.

The CFRP laminates and WWM were used to strengthen the RC columns externally and internally, respectively. The CFRP laminates (Nitowrap CWS 530, Figure 3a) with a thickness of 0.239 mm were used to confine the RC columns. The properties of the CFRP laminates are summarized in Table 2 (from the supplier data sheet). The CFRP was bonded to the RC columns using epoxy resin (Nitowrap Encapsulation Resin) with the properties reported in Table 2. The WWM had 1.1 mm × 2.0 mm openings and 0.6 mm wire diameter (Figure 2). According to ACI-549 [47], the steel of the WWM had yield and tensile strengths of 360 and 420 Mpa, respectively, and 190 Gpa young’s modulus (from the supplier data sheet).

### 2.2. Mix Design and Mixing Procedures

The mixtures’ mixing ratios were designed using the ACI method (Table 3). A control mix (M0), four CRC mixes (Mix1–Mix4), and one concrete mix with RSF (Mix5) were prepared. The crumb rubber was implemented for the CRC mixes to partially substitute the fine aggregate (by volume) with four percentages (10, 20, 30, and 50%). The weight of CR was calculated by multiplying the sand weight by CR/sand specific gravity, or through Equation (1) (the two methods get the same results). The weight percentage of fiber in 1 m^3^ concrete (*W_f_*) is calculated from Equation (1):

(1) $$W_{f}=\frac{V_{f}\times D_{f}}{V_{m}\times D_{m}+V_{f}\times D_{f}}$$

where *V_f_*, *D_f_*, *D_m_*, and *V_m_* are fiber volume fraction, fiber density, matrix density, and matrix volume fraction (*V_m_* = 100 – *V_f_*), respectively. Consequently, one RSF percentage (2.0% by volume fraction of total mix, Mix5) was added to the normal concrete mix to enhance the concrete properties. The aggregates were dry mixed, the cement was added, the aggregates and cement were dry mixed, the water was added to the mixture with a further one minute of mixing, and the CR or RSF was gradually added. One minute more of mixing was achieved. The mixes were used to cast the beams, cubes, and cylinders. The 100 mm cube edge and standard cylinders (diameter = 150 mm and height = 300 mm) were prepared, cured, and tested. The columns, cubes, and cylinders were cured in water for 28 days and then kept in the air at room temperature until testing.

### 2.3. Column Preparation and Details

**Figure 4:**
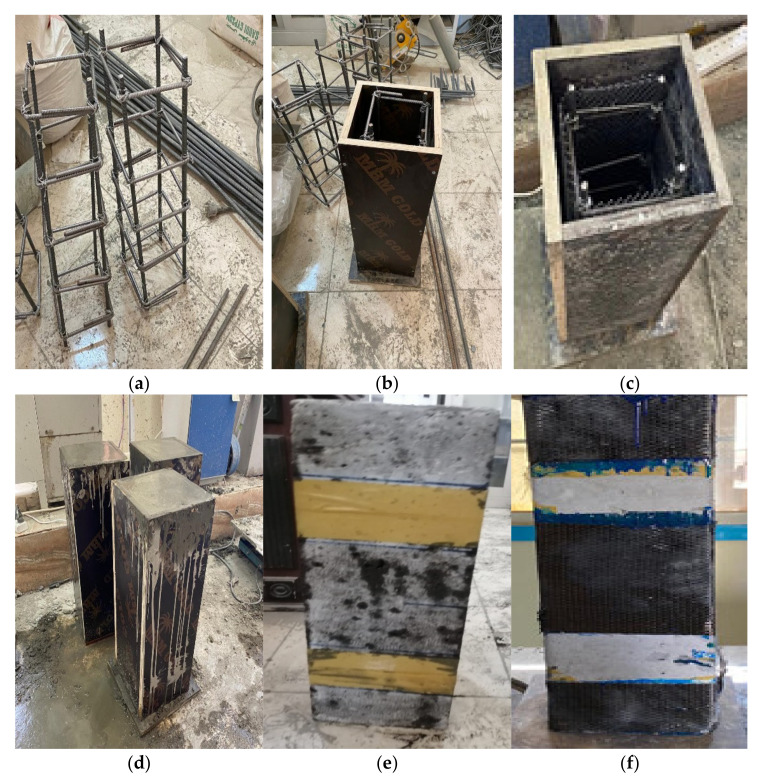
Procedures for column preparation. (**a**) Reinforcement details, (**b**) mold preparation, (**c**) welded mesh application, (**d**) casting, (**e**) surface preparation, and (**f**) CFRP application.

Twenty columns having a 200 × 200 mm cross-section and 600 mm height were prepared and tested (Figure 4). The columns were longitudinally reinforced with four steel bars 10 mm in diameter. Five stirrups 8 mm in diameter were installed to laterally reinforce the RC columns (Figure 4a). The columns were prepared, cured, and tested at room temperature.

**Figure 5:**
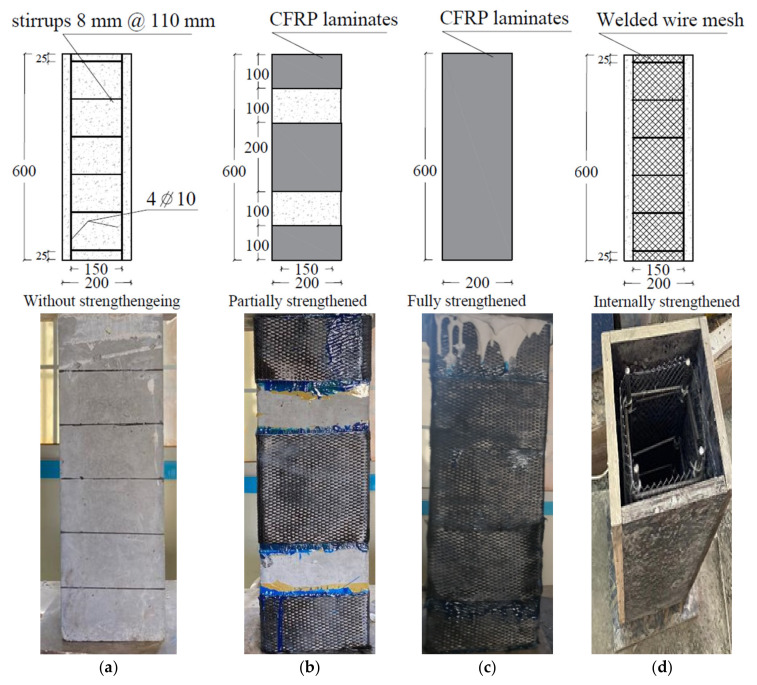
Details of the tested columns. (**a**) Control column, (**b**) partially EB CFRP, (**c**) fully EB CFRP, (**d**) welded wire mesh.

The columns were divided into eight groups depending on the testing variables (Table 4). The concrete type (normal and rubberized concrete), strengthening method (EB CFRP strengthening and internal WWM), strengthening configuration (partial and full EB strengthening), strengthening area (one layer and two layers), and the RSF content (2% RSF) were the considered tested variables. Three columns were prepared from the normal concrete mix (Mix0) (group 1). In this group, one un-strengthened column (Cc) was tested as a control column (Figure 5a), and one column was partially strengthened with one layer of the EB CFRP laminate (Figure 5b) to study the effect of the EB CFRP strengthening on the behavior of the columns. Moreover, the third column was partially strengthened with two layers of the EB CFRP laminates (Figure 5b) to study the effect of the strengthening area on the columns’ behavior.

In group 2, three columns were prepared from a rubberized concrete mix (Mix1 with 10% CR). One un-strengthened column was tested to study the effect of CR content on the columns’ behavior. Conversely, one column was fully strengthened with one layer of the EB CFRP laminates (Figure 5c) to study the effect of the strengthening configuration on the column’s’ behavior. Moreover, the third column was fully strengthened with two layers of the EB CFRP laminates (Figure 5c) to study the effect of the strengthening area on the columns’ behavior.

In two groups (group 3 and group 4), three columns were prepared and strengthened as in group 2, but each group of columns was prepared from a rubberized concrete mix with dissimilar crumb rubber content. The columns located in group 3 and group 4 were prepared from Mix2 (20% CR) and Mix3 (30% CR) to study the effect of CR content on the columns’ behavior. Subsequently, in group 5, two columns were prepared from Mix4 (50% CR); one column was kept without strengthening, and the other was strengthened with two layers of the EB CFRP laminates. In group 6, two columns were prepared from Mix1, one column was internally reinforced with one layer of the SWM, and the other one was strengthened with two layers of the SWM (Figure 5d), to study the effect of the strengthening method on the columns’ behavior.

In group 7, two columns were prepared from Mix2; one column was strengthened with one layer of the SWM, and the other one was strengthened with two layers of SWM (Figure 5d) to examine the influence of the strengthening method and CR content on the behavior of the columns. Finally, in group 8, two columns were prepared, one from Mix5 (2% RSF) and the other from Mix0, to study the effects of RSF and SWM on the columns’ behavior, respectively. The letters and digits of the column identification referred to the test variables (Table 4); the first letter, R, refers to the crumb rubber, the next two digits refer to the crumb rubber content, L refers to the strengthening layer, the next digit (0, 1, or 2) refers to the number of layers, and the last letters (w) refers to the SWM strengthening and (f) refer to the RSF content.

### 2.4. Test Set-Up and Instrumentation

**Figure 6:**
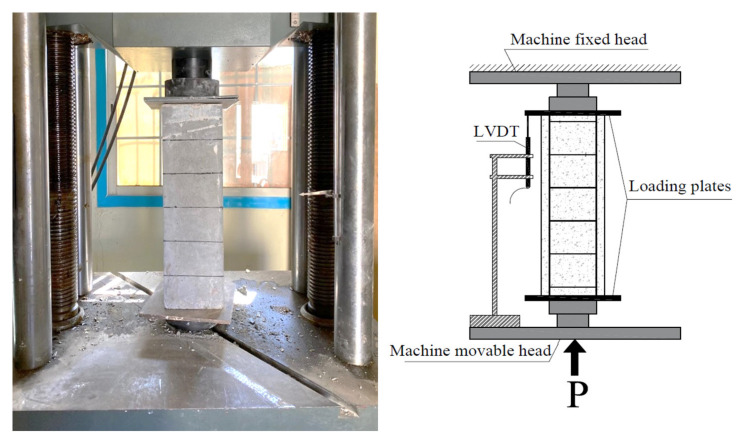
Testing of concrete columns under compression loading.

The hydraulic universal testing machine (200 tons capacity) was used to apply compression loads on the columns (Figure 6). Two steel plates were put on the upper and lower surfaces of the columns to distribute the axial load on the column surfaces. The top surface of the column was smoothed using a Gipson layer before testing. The load was applied gradually on the columns with a displacement rate of 0.5 mm/min until failure. The machine was stopped automatically after the load dropped to 50% of the maximum capacity of the tested column. The column’s contraction was measured using a Linear Variable Differential Transformer (Figure 6). All column data (loads and contraction) were recorded through a computerized system connected to the machine.

## 3. Test Results

### 3.1. Concrete Properties

**Figure 7:**
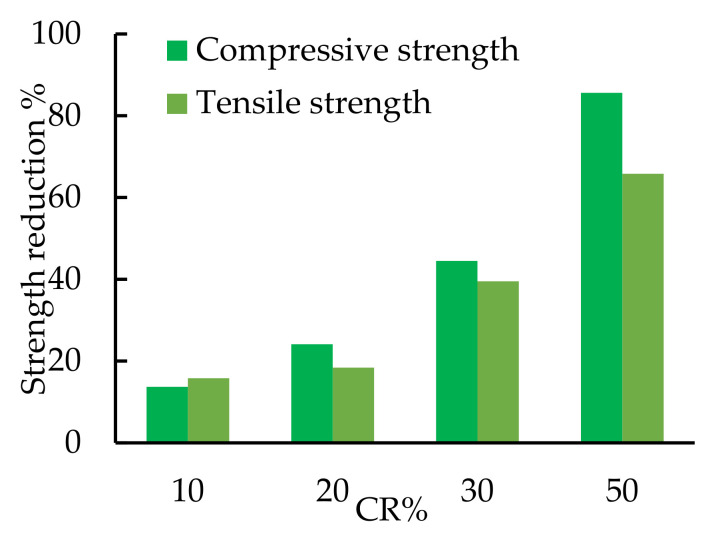
The percentage reduction for both compressive and tensile strengths with CR%.

The properties of the concrete mixes with and without CR are summarized in Table 5. The rubberized concrete’s tensile and compressive strengths (*f_tu_* and *f_cu_*) were reduced as the CR content increased. At the testing date of the beams (60 days from casting), the *f_tu_* and *f_cu_* of Mix0 were 43.1 MPa and 3.8 Mpa, respectively. When the CR replaced sand by 10, 20, 30, and 50%, the compressive strength was diminished by 13.7, 24.1, 44.5, and 85.6%, respectively. In contrast, the tensile strength was reduced by 15.8, 18.4, 39.5, and 65.8%, respectively (Figure 7). The tensile strength reduction was lower than the compressive strength reduction as the CR might help in closing the concrete cracks. Moreover, adding the RSF at 2% of the concrete mix reduced the *f_tu_* and *f_cu_* of this concrete mix by 15.8% and 11.5%, respectively (Table 5). The 2% RSF may be considered a non-suitable content for the concrete as it decreased its strength. Previous studies reported that using 1% or less steel fiber enhanced the concrete properties [48,49,50]. Therefore, more studies are still needed to study the effects of RSF content, both lower and higher than 2%, on the concrete strength.

### 3.2. Column Test Results

#### 3.2.1. Load Capacities and Failure Modes

**Figure 8:**
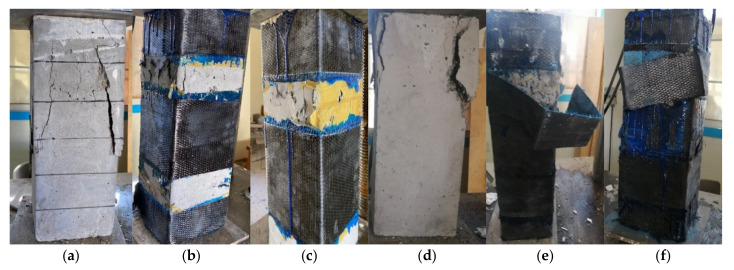

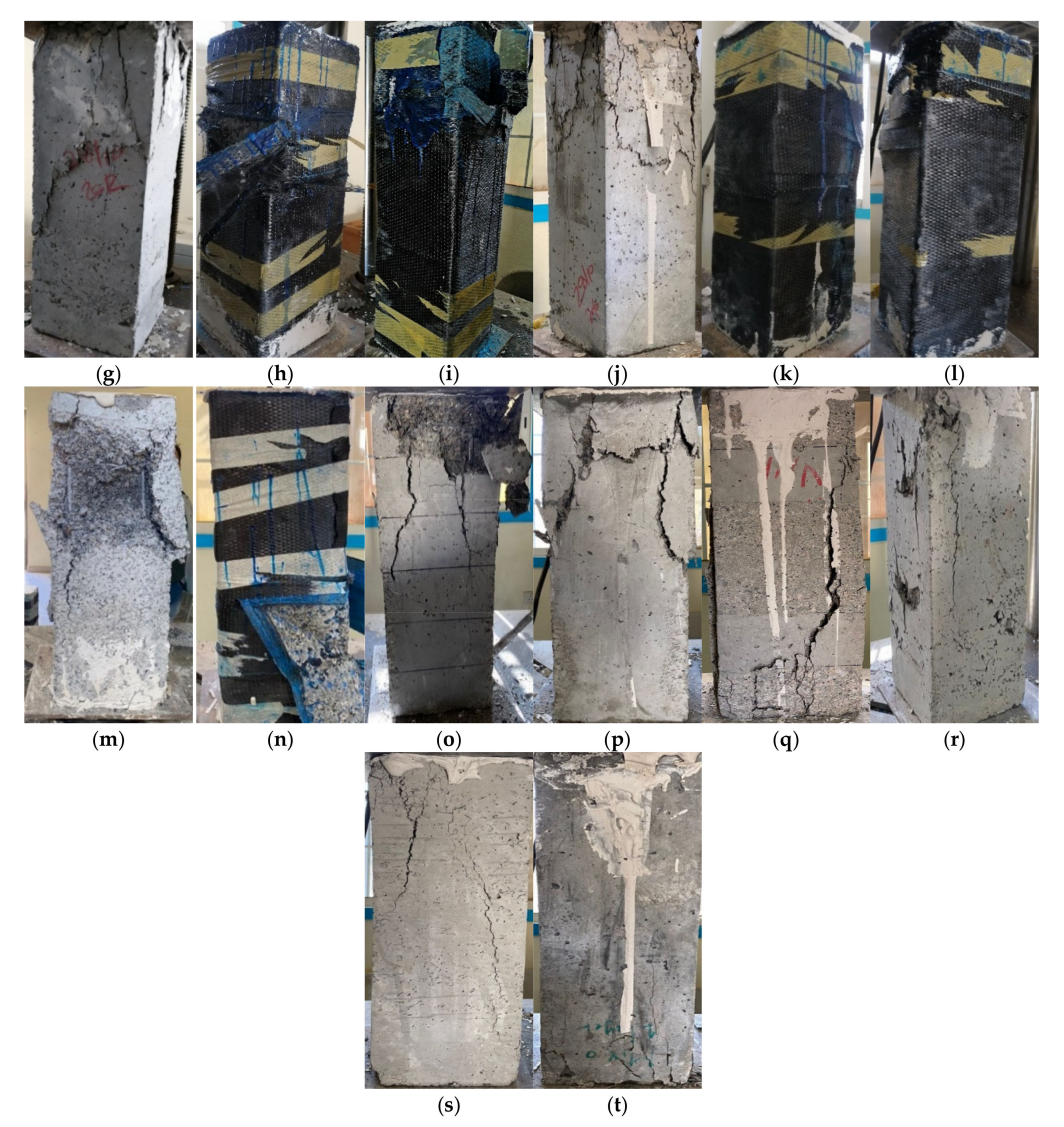
Failure modes of the tested columns. (**a**) R00L0, (**b**) R00L1, (**c**) R00L2, (**d**) R10L0, (**e**) R10L1, (**f**) R10L2, (**g**) R20L0, (**h**) R20L1, (**i**) R20L2, (**j**) R30L0, (**k**) R30L1, (**l**) R30L2, (**m**) R50L0, (**n**) R50L2, (**o**) R10L1w, (**p**) R10L2w, (**q**) R20L1w, (**r**) R20L2w, (**s**) R00L1w, (**t**) R00L0f.

The experimental results of the tested columns in terms of maximum load (*P_u_*) and failure mode are reported in Table 6. The loading efficiency of the columns concerning those with and without CR ($\mu_{u}=\frac{P_{u,str.}}{P_{u,Cc}}$) and the load efficiency of the strengthened columns relative to the corresponding un-strengthened ones ($\mu_{u1}=\frac{P_{ug,str.}}{P_{ug,Cc}}$) are also reported in Table 6. The failure of the un-strengthened specimens cast with normal concrete (R00L0) was concrete crushing (CC) followed by steel buckling (SB) (Figure 8a). In general, all the strengthened columns showed higher maximum load capacities than the un-strengthened columns cast from the same mix. The column strengthened with partially EB CFRP laminates failed due to lateral tension failure (LTF) in the un-strengthened portion (Figure 8b,c). The highest load capacity of the column strengthened with partially EB CFRP laminates was increased by 124.5% compared to the un-strengthened one cast with the same mix. This confirmed the positive effect of the EB CFRP strengthening on the strengthened and un-strengthened portions.

**Figure 9:**
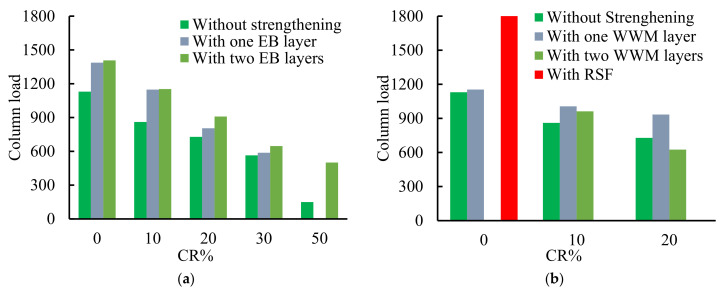
The effect of the EB strengthening, WWM layers, and RSF on the column load. (**a**) Effect of EB layers, (**b**) effect of WWM layers and RSF.

Conversely, doubling the area of the partially EB CFRP strengthening laminates caused a trivial decrease in the column capacity as the failure occurred in the un-strengthened portion (Figure 8c). Consequently, as sand was partially replaced by 10% CR, the load capacity of the tested un-strengthened column decreased by 23.9% compared to the corresponding column cast with mix0 (Figure 9a). The un-strengthened column failed due to concrete crushing (CC) followed by internal steel buckling (SB) (Figure 8a,d). The column partially strengthened with EB CFRP laminates failed due to lateral tensile failure (LTF) of the un-strengthened portion (Figure 8b,c). Moreover, using two EB CFRP layers instead of one layer also had trivial effects on the maximum column load (Figure 9a) as the column failed at the un-strengthened portion. The reason may be the effect of the two layers increasing the lateral stresses transferred to the neighboring un-strengthened portion.

Conversely, the columns strengthened with partially EB layers showed lower load efficiencies than those strengthened with fully EB layers (Table 6), while the failure mode changed from LTF to debonding of the EB CFRP laminates from the concrete (D, Figure 8e), or to debonding of the EB CFRP laminate layers from each other (Ds, Figure 8f). The highest load capacity of the column strengthened with fully EB CFRP laminates was increased by 134.1% compared to that of the un-strengthened one cast with the same mix (Mix1). It could keep the column capacity higher than the un-strengthened column cast with Mix0. This means that the EB strengthening could recover the column capacity lost when using rubberized concrete incorporating 10% CR. Increasing the CR content decreased the column capacity while the EB CFRP effectiveness in confining the RC column increased. The EB strengthening could help to recover a portion of the lost column capacity due to the CR effect (Table 6, Figure 9a).

Columns integrating 20% CR had only 64.5% of the load capacity of the columns cast with normal concrete (NC, Mix0). Increasing CR content to 30% and 50% decreased the capacity of the columns to 50% and 13%, respectively, compared to those cast with NC (Figure 9a). The failure modes of the columns with 20, 30 and 50% CR are shown in Figure 8g–n. Using two layers of the EB CFRP laminates to strengthen the column cast with Mix4 (50% CR) increased the column capacity by 334.3% compared to the un-strengthened column cast with the same mix (the failure was concrete cover splitting, Figure 8n). Contrariwise, the WWM was used to internally strengthen the concrete columns cast with Mix1 (10% CR) in Group 6. The strengthened columns with one and two WWM layers experienced 16% and 11.8% higher load capacities than the same columns without WWM (Figure 9b). Moreover, the columns reinforced with one and two WWM layers could achieve about 89.0% and 85.1%, respectively, of that of column R00L0. Furthermore, the columns cast with Mix2 (20% CR) could attain 82.7% and 55.3%, respectively, of that of column R00L0. The use of two WWM layers decreased the column’s load capacity compared to that with one WWM layer (Figure 9b). This means that increasing the WWM layer might produce voids and defects in the concrete column section and accelerate the concrete cover failure (the column failed due to Cs or LTF, Figure 8o–r). In addition, using the WWM to reinforce the column cast with NC significantly increased its capacity. In contrast, the column cast with NC and 2%RSF experienced higher load capacity than that of R00L0 (about 162.3%), and the failure was CC (Figure 8t).

From all the above results, it was clear that using CR instead of sand decreased the load capacity, but the EB CFRP strengthening and WWM could retain a portion of the load capacity lost in the RC columns cast with CR. Adding RSF to the concrete mix allowed the column to achieve the highest load capacity among all the tested columns, as the column load increased by 162.3% compared to column R00L0. The RSF might increase the column confinement by closing the tensile cracks formed due to lateral tension stresses (Figure 9b).

#### 3.2.2. Load-Contraction Behavior

**Figure 10:**
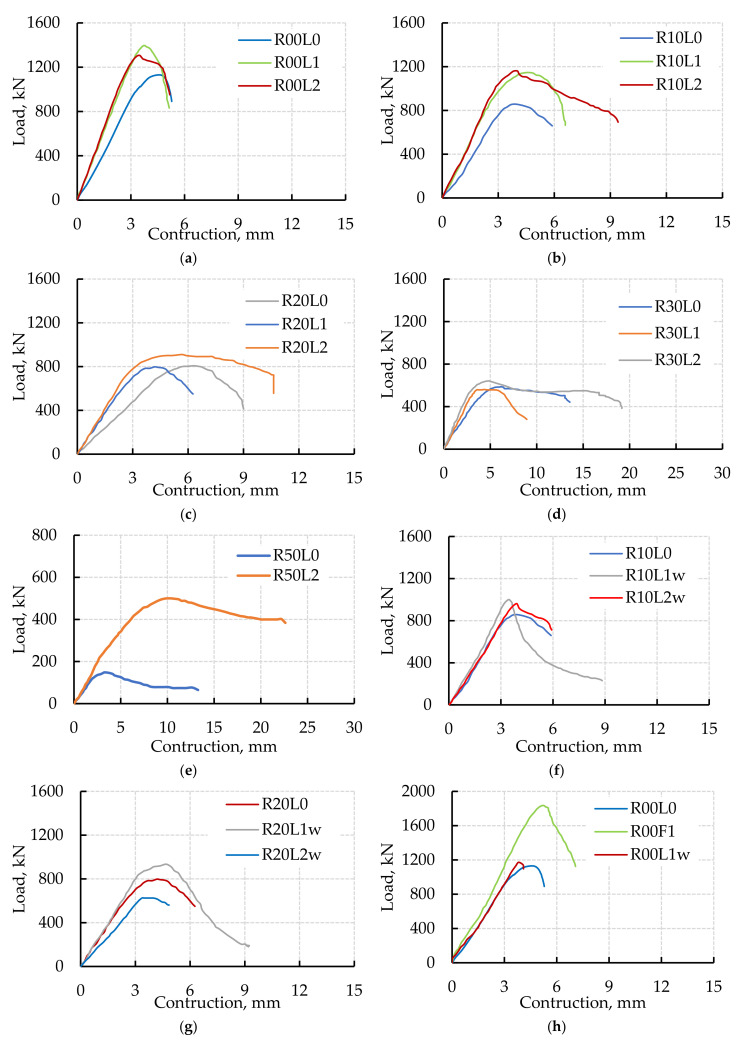
Load contraction curves of the tested columns. (**a**) Columns with Mix0 and CFRP laminates, (**b**) columns with Mix1 and CFRP laminates, (**c**) columns with Mix2 and CFRP laminates, (**d**) columns with Mix3 and CFRP laminates, (**e**) columns with Mix4 and CFRP laminates, (**f**) columns cast with Mix1 and WWM, (**g**) columns with Mix2 and WWM, (**h**) columns with Mix0 and WWM or RSF.

The experimental load-contraction (*P-δ*) behavior of the tested columns is shown in Figure 10. The two partially strengthened columns with EB CFRP laminates showed the same initial stiffness until 75% *P_u_* of the R00L0 column. Moreover, the stiffness of the partially strengthened columns with EB CFRP laminates was higher than the un-strengthened column from the initial loading until failure (Figure 10a). This might be because of the EB laminates’ confinement, which inhibited lateral deformation and diminished column contraction. Conversely, the two columns (R10L1 and R10L2) strengthened with fully EB CFRP laminates showed higher stiffness than the un-strengthened column R10L0 (Figure 10b). This might be because of the effect of CR in decreasing the concrete strength young’s modulus, which led to increasing the lateral deformation and the column contraction. Although increasing the layers of the EB CFRP laminates had little effect on the column load, it enhanced the column’s stiffness until their maximum load. It increased its ductility after the maximum load until reaching its failure load (Figure 10). The replacement of sand by 10% CR increased the ductility of the tested columns compared to the corresponding columns cast without CR.

Moreover, the use of EB CFRP laminates increased the column’s stiffness whatever the CR content (Figure 10b–e). Furthermore, increasing the CR content decreased the column stiffness and increased the column ductility. On the other hand, using one WWM layer or RSF increased the column’s stiffness (Figure 10f–h). In contrast, using two WWM layers decreased the column’s stiffness (Figure 10f–g). Increasing the WWM decreased the column load as the WWM might prevent the concrete from filling all the spaces between the two layers of WWM, increasing the column defects. Finally, using RSF increased both the stiffness and toughness of the column as it decreased the lateral deformation and helped to arrest the column cracking (Figure 10h).

## 4. Discussion and Comparisons

In this section, a discussion of the influence of CR content on the concrete properties and the load capacity of columns compared to the obtained results in this paper is presented. In [51], the reduction in compressive strength was 12.7–26% when the CR replaced the fine aggregate by 5–15%, while in [52] the reduction was 10.9–30.9% when the replacement amount was 6–18%. The strength decreased as the size and content of CR increased, while the concrete ductility decreased [51,52]. In [53], the authors used the same replacement ratios as in [52] but the strength reduction slightly increased to 11.5–31.9%. The strength reduction increased in [53] as the fine aggregate portion increased compared to that in [52]. When the CR replaced the coarse aggregate (CA) by 5–10%, the strength reduction was 10–23%. In [54,55], the strength reduction was 4–70% for the rubberized concrete, when the replacement of the NA by CR was 5–50%. All the previous results confirmed the obtained results reported in the present work. In [24], the strength decreased by 16.1%, 30.5%, and 59.8% as the CR replaced the sand with 5%, 10%, and 20%, respectively. Moreover, treating CR with NAOH could recover part of the lost strength.

The concrete columns strengthened with traditional EB CFRP jackets showed enhancement in their load capacities by 116.2–120.1% compared to corresponding un-strengthened columns [56]. Moreover, the column capacity was also enhanced as the GFRP tube thickness was enlarged, while the concrete strength was raised as the CFRP sheet layers were enlarged [57].

For the tested columns with 200 mm square edge, CFRP strengthening enhanced the load capacity by 117.0–124.8% compared to corresponding columns without strengthening [58]. Moreover, the CFRP confinement decreased as the column size increased [58]. Comparing the present work’s results and previous results for CFRP-strengthened columns showed that the CFRP confinement efficiency is greatly affected by the concrete strength, CR content, internal stirrups, and column size.

**Figure 11:**
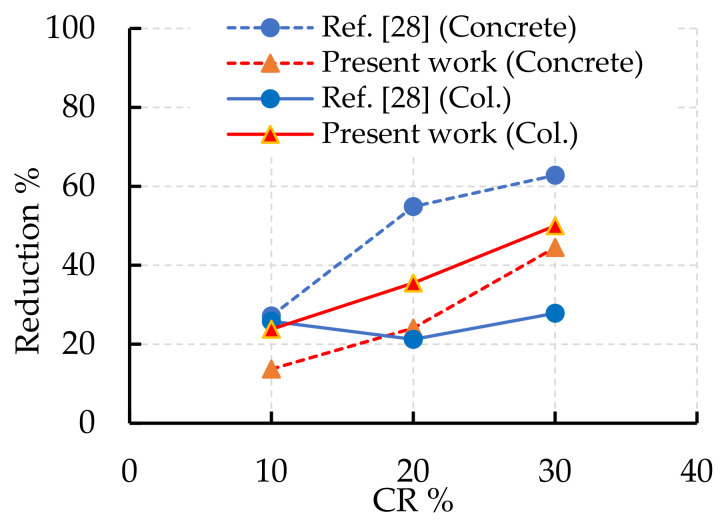
The effect of CR content on the concrete strength and column capacity.

In [28], the natural sand was substituted by 10–30% CR, the concrete compressive strength was obtained, and short steel tube columns were filled using this rubberized concrete. The CR showed a reduction in the concrete strength as the CR content increased. The compressive strength diminished by 27.2%, 54.9%, and 62.8% compared to that without CR [28]. When the previous concrete filled steel tube columns with dissimilar thicknesses, the columns’ load capacity was extremely affected by the tube thickness and the CR content. The steel tube could confine the rubberized concrete inside and decrease the effect of increasing the CR content on the column’s capacity. Loads for 2 mm-thickness filled steel tube columns decreased by 25.8%, 21.2%, and 27.9% for concrete with CR contents of 10%, 20%, and 30%, respectively, compared to normal concrete. The load reductions of the previous column became 0%, 1.6%, and 17.6%, respectively, when the tube thickness was 3 mm, and 7.8%, 14.1%, and 18.8%, respectively, when the tube thickness was 4 mm, when CR contents were 10%, 20%, and 30% compared to those without CR [28]. Conversely, compared to the tested columns in the present work, the CFRP laminates showed a lower effect on confining the concrete columns compared to the filled steel ones (Figure 11). This may be because of the debonding and lower stiffness of the CFRP compared to the steel tubes, and the capability of the steel tubes to carry axial loads in contrast to the EB CFRP laminates. Also, increasing the CFRP laminate thickness (by increasing the CFRP layers) showed slight effects on the column capacity in contrast to the steel tube columns.

## 5. Conclusions

- All the strengthened columns showed higher maximum load capacities than the un-strengthened columns cast from the same mix, while increasing the CR content decreased the load capacities.
- The use of two partially EB CFRP layers instead of one layer decreased the column capacity as it failed at the un-strengthened portion. In contrast, the partially strengthened columns with two layers of the EB CFRP laminates had higher ductility than those strengthened with one EB layer after reaching their maximum load.
- The columns strengthened with fully EB CFRP laminates showed higher stiffness than the un-strengthened columns and the corresponding columns with higher CR content. In addition, doubling the EB CFRP layers increased the column capacity and stiffness until the maximum load, and then increased the column ductility afterward.
- Using one WWM enhanced the column capacity regardless of the CR content, while using two WWMs decreased the column’s load capacity. Conversely, RSF could enhance the column capacity and stiffness as it decreased the lateral column deformation and arrested the column cracking.

In future work, the use of chemical material to enhance the bond between the crumb rubber and the cement mortar to enhance the concrete properties should be studied. Moreover, the use of pozzolanic materials to pre-coat the CR particles to enhance their bond with the cement mortar should be studied.

## Figures and Tables

**Table 1:** Physical properties of natural and crumb rubber.

Physical Properties	Crushed Basalt	Sand	Crumb Rubber
Apparent specific gravity (kg/m^3^)	2.92	2.62	1.26
Bulk specific gravity (SSD) (gr/cm^3^)	2.84	2.28	-
Bulk specific gravity (GD), (gr/cm^3^)	2.80	2.03	-
Water absorption (%)	1.77	2.06	1.03
Moisture content (%)	0.93	1.04	-

**Table 2:** The properties of the CFRP laminates and epoxy (from supplier data sheet).

CFRP Laminates		Epoxy Resin	
Property	Value	Property	Value
Fiber Area Weight, g/m²	530	Specific Gravity	1.1
Ultimate Elongation	2.1%	Pot Life at 20^o^ C, mins	~60
Fiber Strength, Mpa	>4900	Shear Adhesion Strength, Mpa	>14
Fiber Young’s Modulus, Gpa	>230	Compressive Strength, Mpa	>170
Tensile Strength, kgf/cm: width	1050	Compressive Young’s Modulus, Gpa	>2.5
Tensile Strength *, Mpa	3550	Flexural Strength, Mpa	>120
Tensile Young’s Modulus *, Mpa	235 × 10^3^	Tensile Strength, Mpa	>37

* = Design value.

**Table 3:** Mix proportion for 1 m^3^.

Mix ID	CR (%)	W/C (-)	Natural Sand (kg)	Natural Basalt (kg)	Fiber (%)	Water (kg)	Cement (kg)
Mix0	0	0.5	702	1082	-	215	430
Mix1	10	0.5	648	1082	-	215	430
Mix2	20	0.5	576	1082	-	215	430
Mix3	30	0.5	504	1082	-	215	430
Mix4	50	0.5	360	1082	-	215	430
Mix5	0	0.5	702	1082	2.0	215	430

**Table 4:** The columns’ details and test variables.

Group ID	Columns ID	Concrete Mix	Strengthening Layers	Strengthening Scheme	Test Variables
Group 1	R00L0	Mix0	Non	Non	Control column
	R00L1		One CFRP	Partially EB	Strengthening
	R00L2		Two CFRP	Partially EB	Strengthening area
Group 2	R10L0	Mix1	Non	Non	Crumb rubber content
	R10L1		One CFRP	Fully EB	Strengthening configuration
	R10L2		Two CFRP	Fully EB	Strengthening area
Group 3	R20L0	Mix2	Non	Non	Crumb rubber content
	R20L1		One CFRP	Fully EB	
	R20L2		Two CFRP	Fully EB	
Group 4	R30L0	Mix3	Non	Non	Crumb rubber content
	R30L1		One CFRP	Fully EB	
	R30L2		Two CFRP	Fully EB	
Group 5	R50L0	Mix4	Non	Non	Crumb rubber content
	R50L2		Two CFRP	Fully EB	
Group 6	R10L1w	Mix1	One WWM	Fully WWM	Strengthening method
	R10L2 w	Mix1	Two WWM	Fully WWM	No. of steel meshes
Group 7	R20L1w	Mix2	One WWM	Fully WWM	Strengthening method and Crumb rubber content
	R20L2w	Mix2	Two WWM	Fully WWM	
Group 8	R00L0f	Mix5	Non	Non	Recycled steel fiber
	R00L0w	Mix0	One SWM	Fully SWM	Strengthening method

**Table 5:** Compressive and tensile strength of concrete with and without crumb rubber.

Mix, ID	CR, %	*f_cu_*, Mpa	*µ_cu_*, %	*f_tu_*, Mpa	*µ_tu_*, %
Mix0	0	43.1	-	3.8	-
Mix1	10	37.2	86.3	3.2	84.2
Mix2	20	32.7	75.9	3.1	81.6
Mix3	30	23.9	55.5	2.3	60.5
Mix4	50	6.2	14.4	1.3	34.2
Mix5	-	38.6	89.5	3.2	84.2

*f_cu_* = compressive strength, *f_tu_* = tensile strength, *µ_cu_* = ratio of compressive strength of any mix and M0, *µ_tu_* = ratio of tensile strength of any mix and M0.

**Table 6:** Concrete properties and columns’ experimental results.

Group ID	Column ID	*f_cu_* (Mpa)	*µ_1_* %	*f_tu_* (Mpa)	*µ_2_* %	*P_u_* kN	*µ_u1_* %	*µ_u_* %	Failure Modes
Group1	R00L0	43.1	-	3.8	-	1130	-	-	CC, SB
	R00L1					1407	124.5	124.5	LTF
	R00L2					1387	122.7	122.7	LTF
Group2	R10L0	37.2	86.3	3.2	84.2	860.6	-	76.2	CC, SB
	R10L1					1148	133.5	101.6	D, Cs
	R10L2					1153	134.1	102.0	Ds
Group3	R20L0	32.7	75.9	3.1	81.6	728.5	-	64.5	CC
	R20L1					805.0	110.5	71.2	L_f_
	R20L2					909.1	124.8	80.5	Ds
Group4	R30L0	23.9	55.5	2.3	60.5	564.6	-	50.0	CC
	R30L1					587.6	104.0	52.0	CC
	R30L2					647.1	114.6	57.3	CC
Group5	R50L0	6.2	14.4	1.3	34.2	149.8	-	13.3	CC
	R50L2					500.8	334.3	44.3	D, Cs
Group6	R10L1w	37.2	86.3	3.2	84.2	1006.1	116	89.0	Cs
	R10L2w					961.9	111.8	85.1	Cs
Group7	R20L1w	32.7	75.9	3.1	81.6	934.1	128.2	82.7	LTF
	R20L2w					625.3	85.8	55.3	LTF
Group8	R00L1w	43.1	-	3.8	-	1154.0	102.1	102.1	LTF
	R00L0f					1834.3	162.3	162.3	CC

CC = concrete crushing, SB = steel buckling, LTF = lateral tension failure of concrete, D = debonding of the EB laminates from concrete, Ds = debonding of the EB laminate layers from each other, Lf = EB laminates tension failure, Cs = concrete cover splitting.

## Data Availability

The data presented in this article are obtained from an experimental study conducted by the authors.
